# Interventional radiology in the management of vascular complications in urogynecology: a lower-extremity hematoma after transobturator sling—case report

**DOI:** 10.3389/frph.2026.1871761

**Published:** 2026-07-14

**Authors:** Leonardo Gómez Polania, Víctor S. Rangel, Gustavo A. Sarmiento

**Affiliations:** 1 Instituto de la Mujer, Hospital Universitario Mayor-Méderi, Bogotá, DC, Colombia; 2 Universidad del Rosario, Bogotá, DC, Colombia; 3 Clinical Investigation Group, Universidad del Rosario, Bogotá, DC, Colombia

**Keywords:** interventional radiology, lower-extremity hematoma, transobturator sling, urogynecology, vascular complication

## Abstract

**Background:**

Mid urethral slings are currently considered the gold standard surgical treatment for stress urinary incontinence due to their high efficacy and favorable safety profile. Although vascular complications are uncommon, they may occasionally result in clinically significant bleeding.

**Case report:**

We report the case of a 72-year-old woman with stress-predominant mixed urinary incontinence who underwent urethrocolpopexy with placement of a transobturator mid-urethral sling, combined with anterior and posterior colporrhaphy. During the procedure, friable tissue and difficult to control inguinal bleeding were observed. In the immediate postoperative period, progressive inguinal ecchymosis extending to the mons pubis raised concern for a vascular injury, although the patient remained hemodynamically stable, asymptomatic, and without a decline in hemoglobin levels. Given the clinical suspicion, the patient's risk factors, and the technical complexity of the procedure, computed tomography angiography and diagnostic arteriography were performed, both of which excluded active bleeding or vascular lesions. Conservative management and close clinical surveillance led to spontaneous resolution of the hematoma, and the patient was discharged without further complications.

**Conclusion:**

This case highlights a large lower-extremity hematoma as a rare vascular complication associated with transobturator sling surgery and emphasizes the importance of an individualized diagnostic approach. Interventional radiology may serve as a valuable diagnostic and therapeutic tool, allowing exclusion of active bleeding and avoidance of unnecessary surgical reintervention, thereby optimizing patient outcomes.

## Introduction

Stress urinary incontinence (SUI), defined as the involuntary leakage of urine associated with increases in intra-abdominal pressure, is one of the most prevalent pelvic floor disorders in women and is associated with a significant impact on quality of life (1). When conservative management (such as pelvic floor muscle training and even certain pharmacologic treatments) is insufficient, surgical management is considered the treatment of choice (2). For these reasons, mid-urethral slings are currently considered the gold standard surgical treatment for SUI, supported by international clinical guidelines, given their high efficacy (with success rates of approximately 80%–84%) and lower invasiveness compared with traditional techniques such as Burch colposuspension or autologous fascial slings (2).

Surgical management of SUI involves the placement of a synthetic mid-urethral sling to provide support during increases in intra-abdominal pressure. The tension-free vaginal tape, introduced by Petros and Ulmsten in 1996, uses a retropubic approach and was rapidly adopted worldwide. However, concerns regarding vascular and visceral injuries associated with this technique led to the development of the transobturator approach by Delorme in 2001, designed to reduce these risks by directing the tape through the obturator foramen (3). Despite these technical advances and the overall favorable safety profile of suburethral slings, complications may still occur. Among these, vascular injuries are of particular clinical relevance due to their potential to cause significant bleeding.

Hematomas, including retropubic and vulvar types, are recognized complications that may pose diagnostic and therapeutic challenges, particularly when surgical control is limited (4). While small hematomas (<5 cm) are relatively common during the postoperative period, with reported rates of up to 11%, larger and more extensive hematomas are rare. In particular, those extending beyond the pelvic region, such as to the lower extremities, have reported incidences ranging from 0.3% to 0.89% in observational studies (5, 6). These uncommon but clinically significant events are associated with increased postoperative morbidity and are often managed surgically, despite the availability of minimally invasive alternatives such as interventional radiology.

This case report describes a large postoperative hematoma following suburethral sling placement, highlighting the diagnostic challenges and the potential role of interventional radiology in its management. The study was conducted in accordance with the principles of the Declaration of Helsinki. Written informed consent was obtained from the patient. Ethical approval was granted by the Human Research Ethics Committee of Hospital Universitario Mayor–Méderi (Approval No. CEISH-2026009).

## Case presentation

A 72-year-old woman with a history of hypertension, diabetes mellitus, hypothyroidism, prior mediastinal mass resection, and chronic lung disease presented with stage II genital prolapse (POP-Q: Ba +1), stage II posterior compartment prolapse, and mixed urinary incontinence with a stress-predominant component that significantly affected her quality of life. After discussing the therapeutic options, the patient elected surgical management. After obtaining written informed consent, the patient underwent urethrocolpopexy with placement of a transobturator mid-urethral sling, combined with anterior and posterior colporrhaphy.

During the surgical procedure, friable mucosal tissue with a tendency to bleed was observed, likely associated with features of genitourinary syndrome, including vulvovaginal atrophy. When passing the sling using the out–in technique, bleeding occurred in the inguinal region that was difficult to control with direct pressure; therefore, the patient was kept under inpatient observation due to local edema, and additional therapies, including thromboprophylaxis, were deferred.

**Figure 1:**
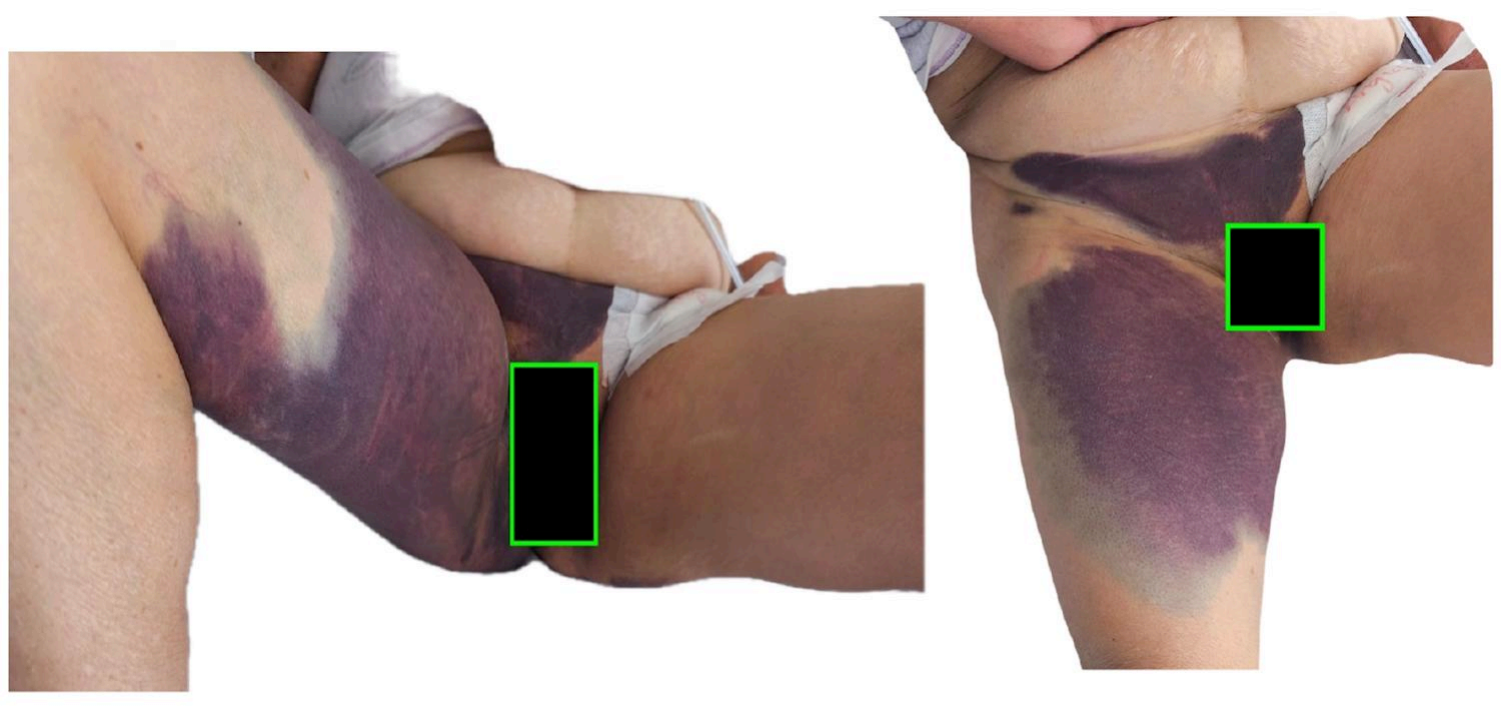
Description of a postoperative-onset hematoma extending to the anterior thigh, gluteal region, and mons pubis.

In the immediate postoperative period, inguinal ecchymosis extending to the mons pubis was documented, with clinical suspicion of progression. Additionally, a hematoma was identified extending caudally to the ischial and gluteal regions and into the right thigh, showing interval expansion compared to the previous day (Figure 1). Nevertheless, the patient remained asymptomatic, with no signs of compartment syndrome or hemodynamic compromise. Vital signs were stable, including a blood pressure of 129/84 mmHg, heart rate of 76 beats per minute, respiratory rate of 16 breaths per minute, and oxygen saturation of 96%. Laboratory results showed stable hemoglobin levels (15.4 g/dL). The leukocyte count was 7,890/mm^3^, hematocrit was 47.7%, and platelet count was 165,000/mm^3^. Renal function parameters were within normal limits, with a blood urea nitrogen level of 12.7 mg/dL and a creatinine level of 0.82 mg/dL.

**Figure 2:**
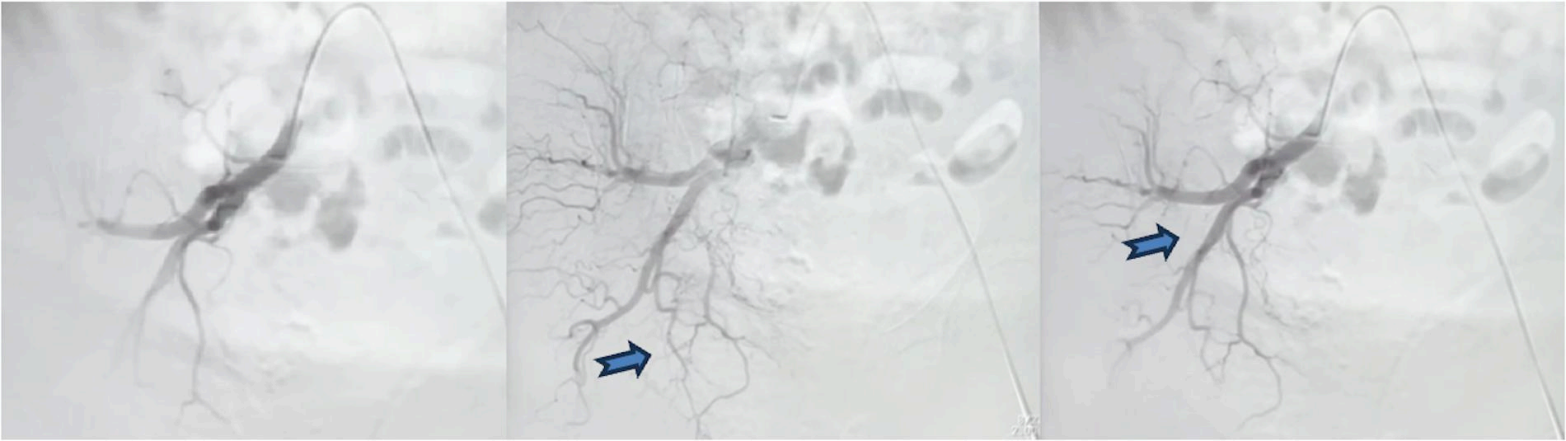
Arteriography of the pelvic and proximal lower-limb arterial vasculature demonstrating preserved vascular integrity, with no evidence of active contrast extravasation. Arrows highlight the evaluated arterial segments, illustrating the absence of contrast leakage or other vascular abnormalities suggestive of an active bleeding source.

Given the suspicion of a vascular injury related to the surgical procedure, and considering the intraoperative findings, the patient's risk profile, and the persistence of clinical concern despite hemodynamic stability, an interventional radiology consultation was requested. Computed tomography angiography was initially performed to characterize the hematoma and assess for pelvic extension that might have warranted initial surgical management via laparotomy. Subsequently, in conjunction with the interventional radiology team, angiographic evaluation was undertaken to determine the presence of active bleeding and the potential need for embolization; no active vascular lesions or ongoing bleeding were identified (Figure 2).

**Figure 3:**
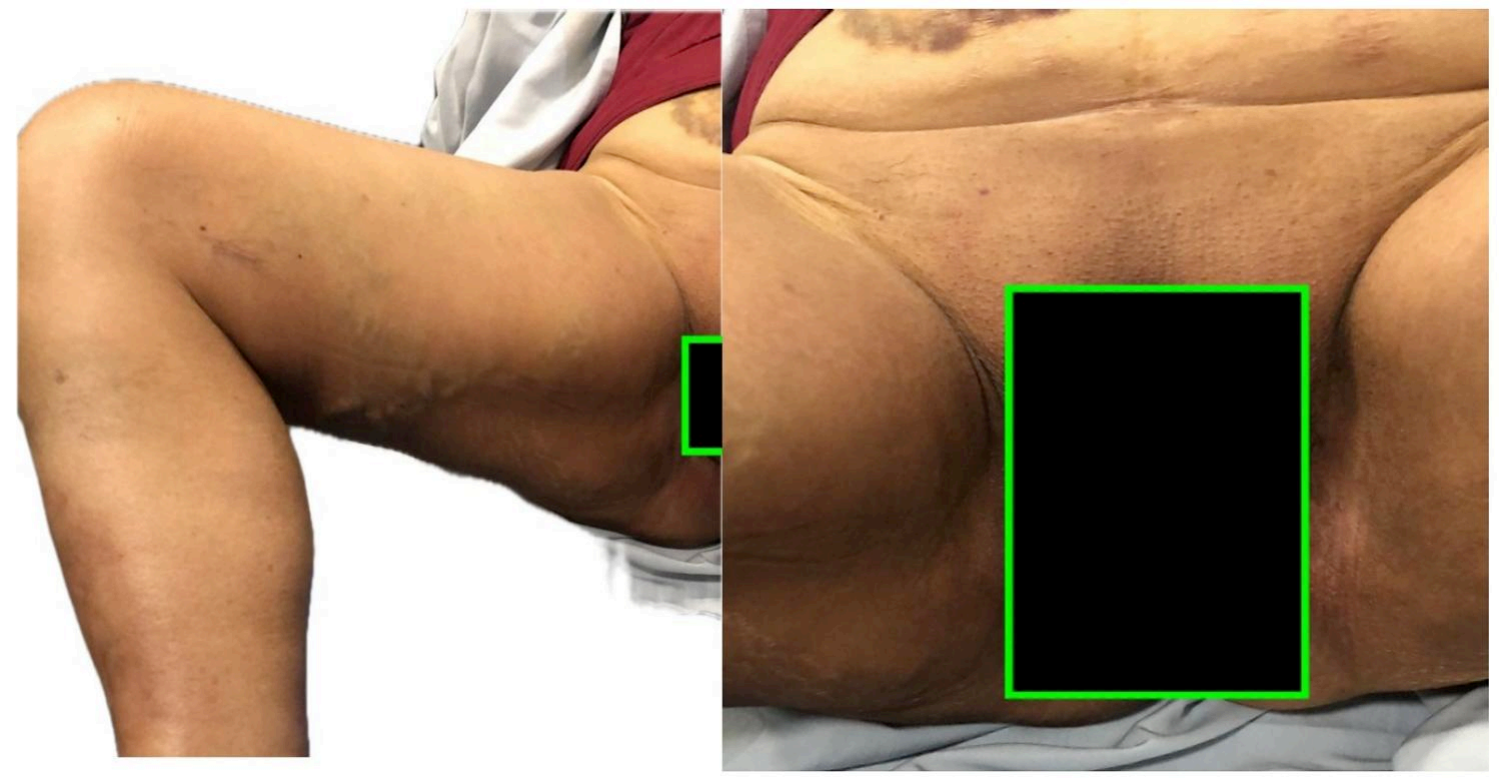
Findings at the 7-day clinical follow-up: no evidence of active ecchymoses, edema, or residual hematomas in the gluteal region, thigh, or mons pubis.

Based on these findings, a conservative management approach with strict inpatient clinical monitoring was adopted. The patient remained hospitalized for 7 days, during which she showed a favorable clinical course, with progressive resolution of the ecchymosis on physical examination. Laboratory parameters remained stable throughout hospitalization, with no evidence of hematologic deterioration or other complications (Figure 3). She was subsequently discharged with thromboprophylaxis, outpatient follow-up, and close clinical monitoring. Follow-up evaluations were performed 7 days and 1 month after discharge, demonstrating continued clinical improvement without evidence of new complications. After the final assessment, the patient was discharged from our institution and continued routine follow-up through her healthcare provider.

## Discussion

Transobturator sling placement follows an anatomical pathway in close proximity to critical vascular and neural structures within the obturator region. During insertion, the device traverses multiple muscular planes, including the gracilis, adductor brevis, and external obturator muscles, as well as the obturator membrane, ultimately passing near the obturator canal (7). From a technical perspective, the trajectory of the needle through the obturator foramen is designed to reduce the risk of retropubic injury; however, it remains closely related to branches of the obturator vessels, which are particularly vulnerable during this approach. On average, the sling lies approximately 1.1 cm from the most medial branch of the obturator vessels (8). This risk is further accentuated in pelvic floor procedures involving deep fixation points, where additional vascular structures, including the internal pudendal vessels and the inferior gluteal artery, may be encountered.

Despite these anatomical considerations, the choice between retropubic and transobturator mid-urethral slings should not be based solely on differences in complication profiles, but also on patient characteristics and surgeon experience. In general, the transobturator approach is often preferred in women with mixed urinary incontinence because it has been associated with a lower risk of *de novo* urgency symptoms and worsening of pre-existing urgency. Additionally, by avoiding passage through the retropubic space, the transobturator approach reduces the risk of visceral injuries, particularly bladder and bowel injuries, as well as major vascular complications, making it a widely adopted surgical option. Nevertheless, although major vascular complications are uncommon, they remain possible because of the close anatomical relationship between the transobturator trajectory and the vascular structures described above (3, 9).

In this context, bleeding is a recognized complication of suburethral sling surgery, with a clinical spectrum ranging from minor hemorrhage during periurethral dissection (generally self-limited and controllable with direct compression) to major vascular injury with the potential for hemodynamic instability and serious outcomes (3, 10). The therapeutic approach depends on the magnitude and clinical impact of the bleeding. In most mild to moderate cases, conservative management (consisting of vaginal packing and timely completion of the surgical procedure) is sufficient to achieve hemostasis. Immediate surgical exploration is reserved for significant bleeding or hemodynamic instability, whereas in carefully selected cases, endovascular embolization may be considered an effective option for managing major vascular injuries or persistent hemorrhage (11).

Interventional radiology represents a valuable therapeutic option in the management of hemorrhagic complications associated with suburethral sling surgery, particularly in patients with severe or persistent bleeding in whom conventional measures are insufficient or entail a high surgical risk. Selective arterial embolization allows precise identification and occlusion of the bleeding vessel through minimally invasive techniques, achieving effective hemostatic control while avoiding open surgical reintervention.

A case report described by Ferraz et al. documented a pelvic hematoma following a transobturator sling procedure in the context of clear hemodynamic instability, with active vascular bleeding requiring urgent embolization. In that case, the hemorrhagic involvement was confined to the intrapelvic compartment, underscoring the importance of early imaging (particularly computed tomography) to guide initial management and identify patients who may require surgical or endovascular intervention (12). In contrast, our case presented a different clinical scenario, characterized by hemodynamic stability despite a high risk of hemorrhagic complications and, notably, by extrapelvic extension of the hematoma into the lower extremity. These findings reflect the presence of different patterns of vascular involvement, which may complicate the initial clinical assessment. In this context, management should prioritize the exclusion of intrapelvic vascular injury, given its higher risk of morbidity and mortality, before addressing extrapelvic involvement.

Similarly, Kyung et al. reported a case of vaginal hematoma following a transobturator sling procedure, in which bleeding from the internal pudendal artery required embolization to achieve hemostasis. In that case, the hematoma remained confined to the vaginal compartment, a feature that may partially obscure early clinical manifestations and delay recognition of the underlying vascular injury. Despite initial surgical management, persistent bleeding necessitated endovascular intervention (13). From a clinical perspective, a thorough physical examination should be prioritized prior to any interventional approach, as pelvic involvement remains the most common clinical presentation of these hematomas, highlighting the rarity of extrapelvic extension.

Conservative management has been described in similar cases in the literature. For example, Yavaş Yücel et al. reported a case of a thigh hematoma involving the medial aspect of the thigh following a transobturator sling procedure, successfully managed with clinical monitoring and supportive measures without the need for invasive intervention (14). However, unlike our case, no interventional radiology–based approach was performed. Angiographic evaluation plays a key role in this context, not only in confirming the diagnosis but also in excluding active vascular injury and guiding management decisions, including the need for interventions such as embolization. Although the use of interventional radiology in this setting remains uncommon and is largely limited to case reports, available evidence suggests it is a safe and effective alternative to surgical exploration, particularly for lesions that are difficult to access or in the presence of extensive hematomas (11, 15).

**Figure 4:**
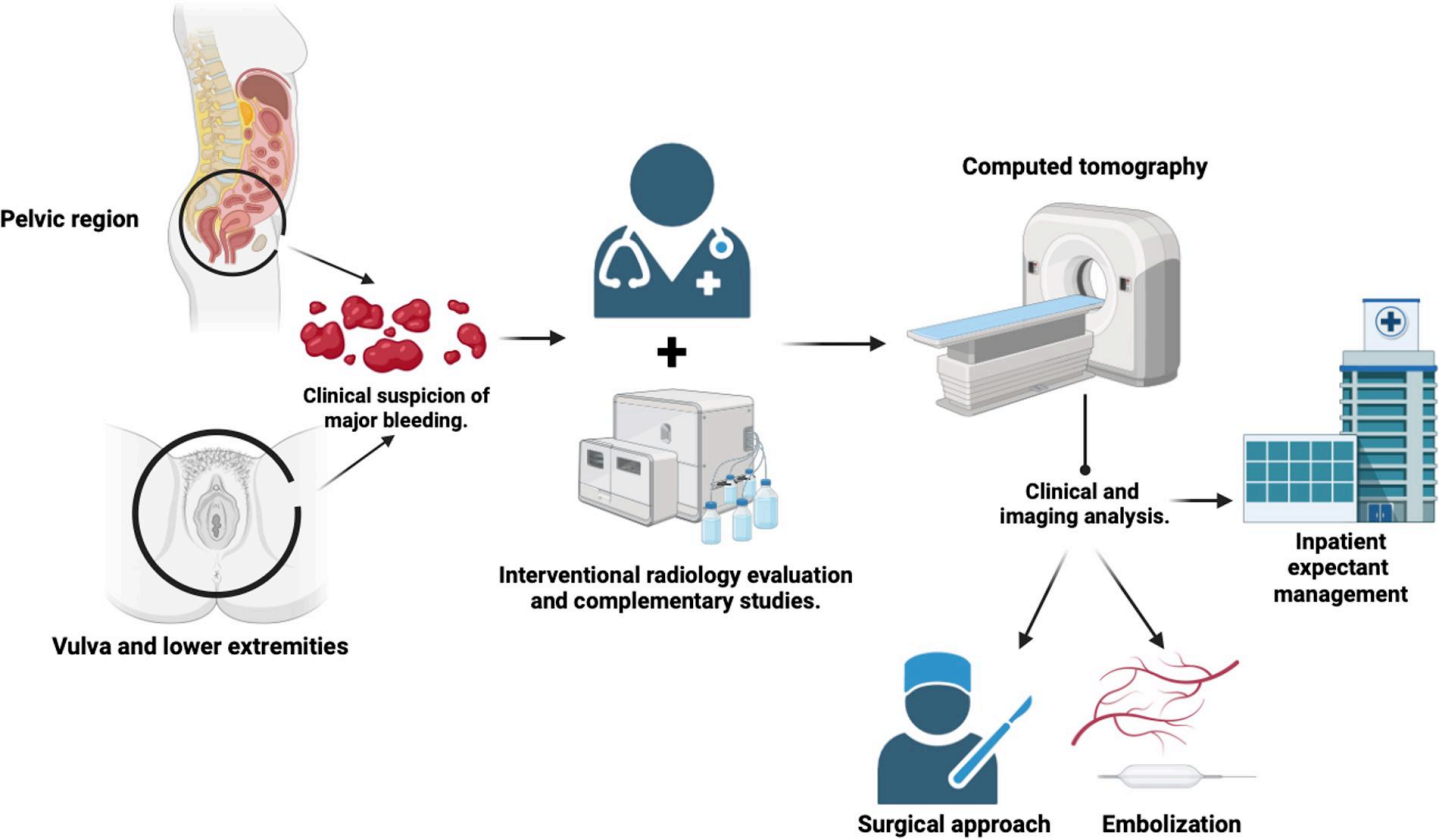
Proposed clinical algorithm for the evaluation and management of suspected major vascular complications after urogynecologic procedures, emphasizing a stepwise approach based on clinical assessment, imaging findings, and interventional radiology to guide therapeutic decision-making.

In our case, the integration of imaging findings and interventional radiology assessment enabled a more comprehensive and individualized approach, ultimately supporting conservative management under close clinical monitoring. Based on the available literature and the multidisciplinary experience described in this report, we present a proposed framework for the evaluation and management of suspected major vascular complications following urogynecologic procedures (Figure 4). However, given the limited evidence available and the rarity of these complications, this framework should be interpreted with caution and not as a validated management algorithm. Further studies are needed to evaluate the potential benefits, risks, and clinical impact of incorporating interventional radiology into the diagnostic and therapeutic pathways of these patients.

## Conclusion

Interventional radiology may serve as a valuable diagnostic adjunct in the evaluation of suspected hemorrhagic complications following suburethral sling procedures. In this case, angiographic assessment excluded active bleeding or vascular lesions, avoiding unnecessary intervention. The patient had a favorable clinical course, with spontaneous resolution of the hematoma and no subsequent complications. These findings suggest a potential role for interventional radiology in selected patients; however, conclusions derived from a single case should be interpreted with caution, given the inherent limitations in generalizability. Further studies are required to better define its clinical utility and to establish standardized criteria for its use.

## Data Availability

The original contributions presented in the study are included in the article/Supplementary Material, further inquiries can be directed to the corresponding author.
